# Prolonged Response of Metastatic Programmed Death-Ligand 1 (PD-L1) Negative Basaloid Squamous Cell Carcinoma of the Lung to Maintenance Immunotherapy

**DOI:** 10.7759/cureus.24396

**Published:** 2022-04-22

**Authors:** Ashley D Fox, Asad Ullah, Lakshmi K Vemavarapu, Freyli Bustamante, Nagla Abdel Karim

**Affiliations:** 1 Internal Medicine, Medical College of Georgia, Augusta University, Augusta, USA; 2 Pathology, Medical College of Georgia, Augusta University, Augusta, USA; 3 Pathology, Veterans Affairs Medical Center, Augusta, USA; 4 Radiology, Medical College of Georgia, Augusta University, Augusta, USA; 5 Hematology and Oncology, Georgia Cancer Center, Augusta University, Augusta, USA

**Keywords:** metastatic non-small cell lung cancer, cancer-immunotherapy, lung cancer, basaloid squamous cell carcinoma, basaloid carcinoma of lung

## Abstract

Basaloid squamous cell carcinoma (BSCC) is a variant of squamous cell carcinoma that is most often seen as a variety of primary head and neck cancers. BSCC of the lung is rare primary lung cancer and an uncommon histological subtype of non-small cell lung carcinoma. Due to its rarity, there is little published information and research available to direct disease-specific management of BSCC of the lung. Most published cases of basaloid carcinoma of the lung report on surgical management of eligible patients and even less information can be found for metastatic cases. We report a case of a 74-year-old male with stage four (IV) BSCC of the lung who experienced a complete metabolic with partial anatomic response to combined chemotherapy and immunotherapy with carboplatin/nab-paclitaxel/pembrolizumab and has continued to be in partial remission on maintenance immunotherapy with pembrolizumab despite PD-L1-negative status.

## Introduction

Basaloid squamous cell carcinoma (BSCC) of the lung is a rare subtype of squamous cell lung carcinoma. BSCC accounts for an approximated 3.9-5.2% cases of all squamous cell lung cancers [1]. It has been associated with rapid clinical progression, rapid growth rate, and a poorer prognosis compared to conventional squamous cell carcinoma of the lung. Furthermore, it has been suggested that high expression of programmed death-ligand 1 (PD-L1) in BSCCs is associated with better outcomes, including disease-free and overall survival, for this cancer [2]. This might indicate that PD-L1 negative status would confer an even more dismal prognosis in BSCC. The optimal treatment for basaloid squamous cell lung cancer remains unknown and more data is needed in this group of patients. Our case demonstrates an exceptional response of stage four (IV) PD-L1 negative (0%) basaloid squamous cell lung cancer to platinum-based chemotherapy plus immunotherapy regimen.

## Case presentation

**Figure 1:**
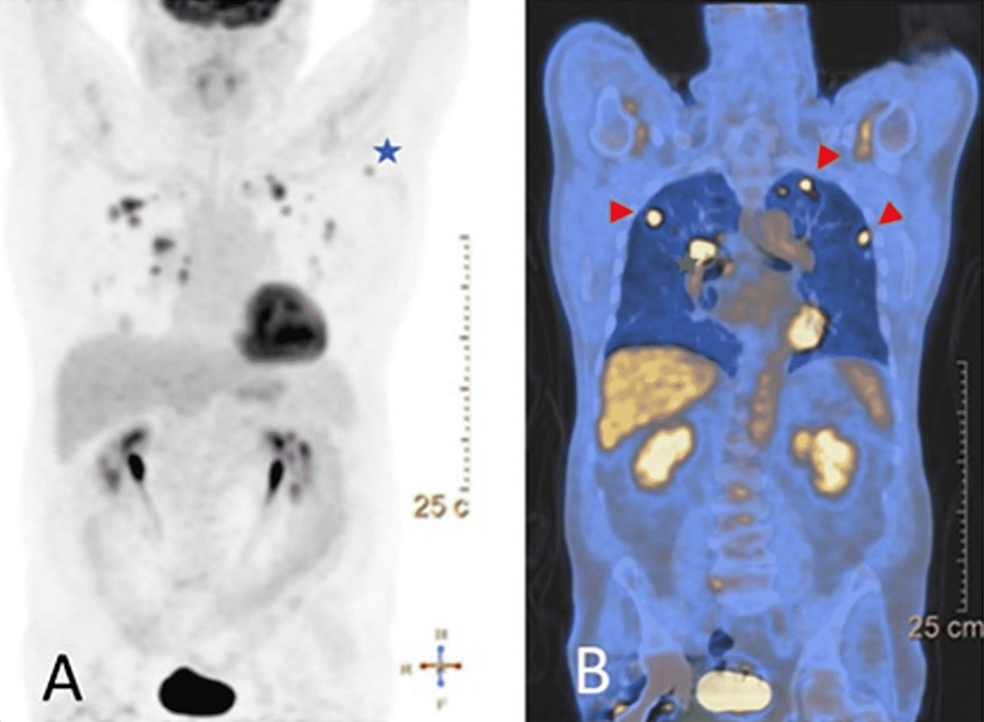
Initial positron emission tomography–computed tomography (PET/CT) findings. A) maximum intensity projection (MIP) (Blue star: hypermetabolic axillary lymphadenopathy). B) coronal-fused PET/CT demonstrates multiple bilateral hypermetabolic pulmonary nodules, intense fluorodeoxyglucose (FDG) avid right hilar metastatic lymphadenopathy, and metastatic hypermetabolic left axillary lymph node (Red arrowhead: hypermetabolic pulmonary nodules, Green star: hilar metastatic lymphadenopathy).

A 74-year-old Caucasian male was referred to our clinic after a chest X-ray obtained for workup of a dry cough persisting for approximately three months revealed three lung nodules. Initial positron-emission tomography (PET) scan revealed multiple (more than five) hypermetabolic nodules within all lobes of the left and right lungs. The largest nodule in the right lung measured 1.5 x 1.7 cm with a standardized uptake value (SUV) of 8.2 and the largest within the left lung measured 1.7 x 2.7 cm with an SUV of 8.6. The PET scan also demonstrated multiple hypermetabolic hilar lymph nodes, the largest of which measured 1.4 x 0.5 cm with SUV of 7.9, mildly hyperbolic left hilar lymphadenopathy, and a hypermetabolic left axillary lymph node representing metastatic lymphadenopathy (Figure 1).

**Figure 2:**
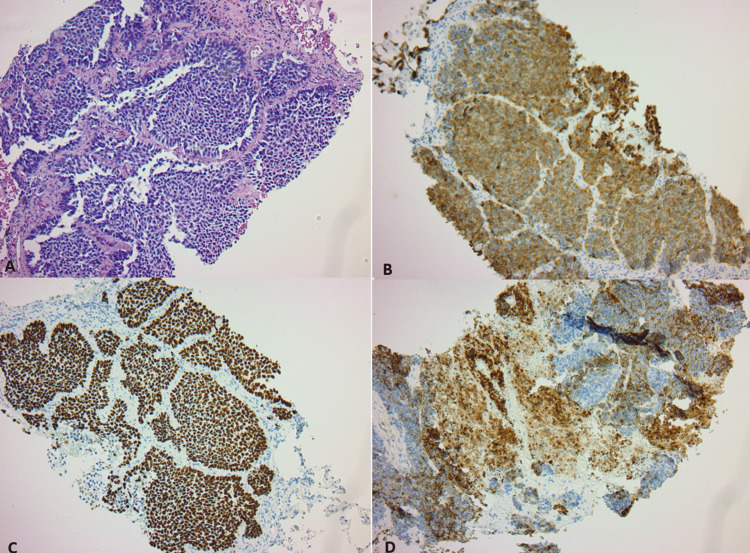
Immunohistochemical staining of the lesion. A) hematoxylin and eosin (H&E) stain 20×, sheets of dark round blue, basophilic cells with minimum cytoplasm with peripheral palisading and fibrotic stroma. B) Pankeratin stain reveals strong cytoplasmic staining of tumor cells. C) P40, showing diffuse and strong nuclear staining of tumor cells. D) Cytokeratin 7 (CK7): demonstrating focal staining of tumor cells.

The patient underwent a biopsy of one of the right apical lung nodules which revealed sheets of hyperchromatic basaloid cells with minimal cytoplasm and peripheral palisading separated by fibrous bands. Immunohistochemical staining of the lesion showed tumor cells were strongly positive for pankeratin, cytokeratin 7 (CK7), P40, and high nuclear protein Ki67 (Ki67) proliferation index (Figure 2). The tumor cells were negative for synaptophysin, chromogranin, neural cell adhesion molecule CD56 (CD56), and thyroid transcription factor-1 (TTF-1). The expression of PD-L1 was 0%. At the time of diagnosis, the clinical staging was M1a stage IV.

**Figure 3:**
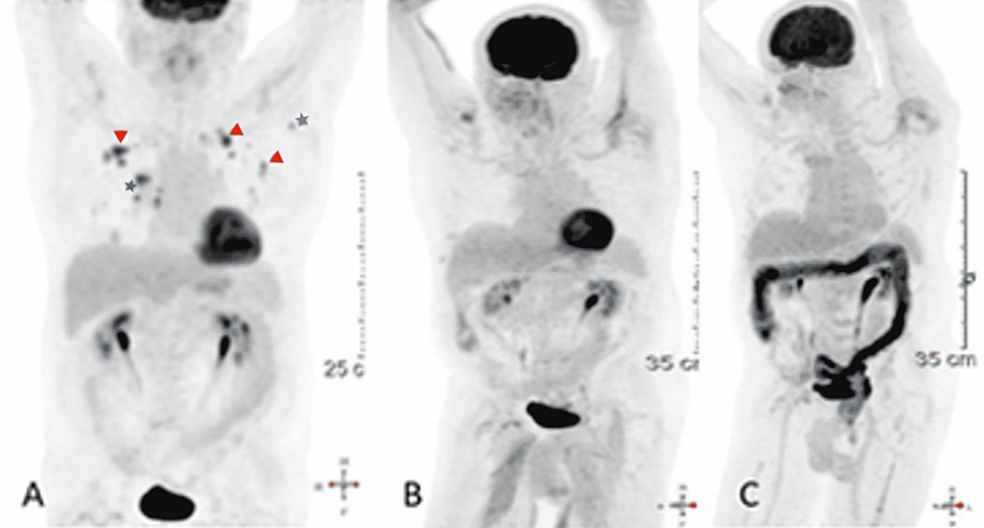
PET scan after cycles 15 and 16 of the adjusted regimen. A) Initial positron-emission tomography–computed tomography (PET/CT), maximum intensity projection (MIP), demonstrates multiple bilateral hypermetabolic pulmonary nodules, intense fluorodeoxyglucose (FDG) avid right hilar metastatic lymphadenopathy, and metastatic hypermetabolic left axillary lymph node (Red arrowhead: hypermetabolic pulmonary nodules, Green star: hilar metastatic lymphadenopathy, Blue star: hypermetabolic axillary lymphadenopathy). B) MIP image, after completion of five cycles of carboplatin/nab-paclitaxel/pembrolizumab. C) MIP images after 16 cycles of pembrolizumab show resolution of previous hypermetabolic activity of multiple bilateral pulmonary nodules, consistent with response to treatment; also incidentally showed diffuse inflammatory bowel activity.

The patient received four cycles of carboplatin/nab-paclitaxel/pembrolizumab. After just two cycles, the PET scan demonstrated an interval decrease in the size of multiple nodules, no remaining metabolically active nodules, and resolution of hypermetabolic lymph nodes. Following completion of four cycles, a repeat PET scan showed continued decrease in size of nodules and resolution of hypermetabolic activity of pulmonary nodules with no metabolically active tumor, resolution of bilateral hilar lymphadenopathy, and no evidence of distant metastasis. The patient was then started on maintenance immunotherapy with pembrolizumab. He received pembrolizumab 200 mg every three weeks for 14 cycles. At this time, surveillance PET scan demonstrated continued complete metabolic remission, his pembrolizumab dose was changed to 400 mg every six weeks. Repeat surveillance PET scan after cycles 15 and 16 of the adjusted regimen showed continued complete metabolic response of bilateral pulmonary nodules and no new nodules or evidence of active nodal disease or distant metastasis (Figure 3). The patient has now completed two years of maintenance therapy with pembrolizumab.

## Discussion

It has been reported that basaloid lung carcinoma has a poorer prognosis than other non-small cell lung cancers (NSCLC) and squamous lung carcinomas [2]. Previous reports have suggested that patients with basaloid squamous cell lung cancer have a median survival of 20-29 months even in patients with stage I and II resected tumors [3]. In comparison, our patient, diagnosed with stage four (IV) disease, received no surgical intervention and achieved complete metabolic remission and partial anatomic response to four cycles of carboplatin/nab-paclitaxel/pembrolizumab. Furthermore, after our patient was initiated on maintenance therapy with pembrolizumab, he continued to have an anatomic response with continued decrease in size of the pulmonary nodules. To date, our patient has no remaining metabolically active tumor, representing maintenance of partial remission and progression-free survival of over 24 months. To the best of our knowledge, this is the first report of such an exceptional response of a PD-L1 negative basaloid squamous cell lung cancer to this treatment regimen.

At present, the standard therapy for squamous cell lung cancers is also applied to basaloid squamous cell lung cancers. The phase-3 KEYNOTE-407 trial demonstrated that the addition of the PD-1 inhibitor pembrolizumab to standard chemotherapy with carboplatin and either paclitaxel or nab-paclitaxel led to significantly longer overall survival and progression-free survival in patients with metastatic squamous NSCLC of any level of PD-L1 expression [4]. However, it was noted that greater PD-L1 expression was associated with longer overall and progression-free survival. In this study, the median progression-free survival among patients with PD-L1 negative status receiving platinum-based chemotherapy plus immunotherapy was 6.3 months while mean overall survival was 15.9 months. Our patient has demonstrated a more exceptional response in comparison to these data.

In another review, it was reported that 15% of patients with PD-L1-negative tumors of varying cancer types had a positive response rate to PD-L1 targeted therapy, compared with a 48% response rate for patients with PD-L1-positive tumors [5]. Comparing our patient’s response to immunotherapy, it is possible that patients with PD-L1 negative BSCC may demonstrate a greater response to immunotherapy compared to other types of squamous cell lung cancer despite its overall poorer prognosis.

## Conclusions

Basaloid carcinoma of the lung is a rare type of primary lung cancer that carries a poor prognosis and does not have disease-specific treatment guidelines. We observed a favorable response of metastatic BSCC of the lung to maintenance immunotherapy following platinum-based chemotherapy in spite of negative PD-L1 expression. This case supports that the use of platinum-based chemotherapy can achieve excellent metabolic and anatomic outcomes in patients with metastatic basaloid carcinoma of the lung. In addition to the response to the standard platinum-based chemotherapy, the patient presented in this case experienced ongoing anatomic response during maintenance therapy with immunotherapy. This was evidenced by a continued decrease in tumor size throughout this stage of treatment. Furthermore, complete metabolic remission on immunotherapy was maintained for a prolonged period despite PD-L1 negative status.

Our patient’s combined ongoing anatomic response and maintained complete metabolic remission to maintenance immunotherapy over the course of two years may suggest that PD-L1 negative basaloid carcinomas of the lung may respond more favorably to PD-L1 targeted immunotherapy than other cancer types. Therefore, investigations should be carried out to evaluate the specific clinical response of basaloid carcinoma of the lung, particularly metastatic disease, to immunotherapy which may guide disease-specific treatment recommendations to optimize the management of this rare lung cancer.
